# Exploring the Effect of Yoga on Exercise Endurance As Assessed by Cardiorespiratory Efficiency Tests in Exercise Physiology Laboratory: A Pilot Study

**DOI:** 10.7759/cureus.38283

**Published:** 2023-04-29

**Authors:** Ruchi Kothari, Gaurav Mittal, Prashanth A, Pradeep Bokariya

**Affiliations:** 1 Department of Physiology, Mahatma Gandhi Institute of Medical Sciences, Wardha, IND; 2 Department of Anatomy, Mahatma Gandhi Institute of Medical Sciences, Wardha, IND

**Keywords:** medical student assessment, cardiorespiratory fitness, physical ﬁtness index, harvard step test, breath-holding test, yoga research, vo2 max

## Abstract

Background

Today's world of cut-throat competition is boggling with stress as the most common problem among the modern generation, and reduction in stress demands a radical solution. Yoga comes as a rescuer that focuses on improving one's physical and spiritual well-being. It can increase one's strength and flexibility. Yoga practitioners have asserted the effect of physical exercise involved in it on balancing physical and spiritual health for decades, but only recently has there been a move to substantiate these claims through research. This study aimed at assessing the effect of yogic practice on exercise endurance and physical fitness as assessed by important physical fitness parameters through cardiorespiratory efficiency tests in an Exercise Physiology Laboratory.

Methodology

A total of 60 Bachelor of Medicine, Bachelor of Surgery (MBBS) students from a rural medical college in central India were recruited for the study. Thirty MBBS students who had undergone yogic training for six months comprised the trained or the case group, and another group of 30 students comprising the untrained group were recruited for the study from different levels of the course within the age group of 17-25 years. Body mass index (BMI) and body surface area (BSA) were calculated. Resting pulse rate and blood pressure, resting respiratory rate, maximal oxygen consumption (VO_2_ max), physical ﬁtness index (PFI), breath holding time (BHT), and 40 mm Hg endurance test time was measured.

Results

The mean PFI (%) in males was 88.82±5.56 and 96.05±7.44, and that in females was 82.06±8.95 and 96.55±6.47 in the control and case groups, respectively. The mean 40 mm Hg endurance test (in seconds) in males was 36.47±8.45 and 48.88±8.64 and in females was 29.79±10.30 and 38.4±10.69 in the control and test groups, respectively. The mean BHT (in seconds) in males was 44.80±14.18 and 58.91±12.35, and that in females was 42.29±15.37 and 54.60±13.36 as in control and case groups, respectively. The VO_2_ max evaluated by the modified Harvard step test was 2.41±0.58 L/min in control males and 3.6±0.90 L/min in the case group of males, and it was 2.14±0.49 L/min in the control group of females, and 3.76±0.69 L/min in case group of females.

Conclusion

By studying the dynamics of the various cardiorespiratory responses, we have determined the values of fitness parameters in the case group. It was found that the yoga group had statistically significantly higher VO_2_ max per minute and better PFI, BHT, and 40 mm Hg endurance values (p<0.05).

## Introduction

Cardiovascular disease is becoming one of the leading causes of death globally. One primary prevention for it could be physical activity in the form of yogic practice [1]. Yoga focuses on improving your physical, mental, and spiritual well-being. The goal of yoga is to harmonize your body, mind, and spirit through a combination of poses, meditation, and breathing exercises. Practicing yoga has many physical and mental benefits. For example, the physical exercise involved in yoga can increase your strength and flexibility. Yoga poses are generally done with deep, diaphragmatic breathing that is thought to increase oxygen flow to the brain [2]. Yoga practitioners have asserted its effect on balancing emotional, physical, and spiritual health for decades, but only recently has there been a move to substantiate these claims through research. So far, the result has been definitive, yet there is meager evidence of the far-reaching benefits of yoga, both as a treatment and as a preventative form of health care.

Yoga is rapidly gaining popularity as the number of people practicing yoga for health beneﬁts in India as well as abroad has increased signiﬁcantly in the past decade. In spite of the continued interest of the scientiﬁc community, there is still a paucity of data on basic physiological responses related to yoga practices. The data on cardiorespiratory parameters in those who practice yoga, as well as relevant data to express the intensity of exercise in terms of exercise physiology, have not been documented systematically.

The major objective of the study was to determine the impact of yoga practice on physical fitness based on the assessment of cardiorespiratory efficiency in the Exercise Physiology Laboratory. Hence this pilot study would, in turn, help evaluate the baseline effect of yoga on cardiorespiratory pursuits in individuals already practicing yoga and hence help speculate a proper intervention plan in order to study the effect of a yogic regime on physical finesse.

## Materials and methods

Study design and setting

It was a cross-sectional case-control study. The Strengthening the Reporting of Observational Studies in Epidemiology (STROBE) guidelines for case controls were used for reporting and preparing the manuscript. The study was carried out in the Exercise Physiology Laboratory of the Department of Physiology of a rural medical college within a duration of six months.

Study participants

Sixty Bachelor of Medicine, Bachelor of Surgery (MBBS) students of a rural medical college located in central India were included in the study from different levels of the course within the age group of 17-25 years. It was a pilot study in accordance with a bigger project with a high sample size; a 10% size was used, which came out to be 60 subjects. Amongst the 60 recruited subjects, 30 participants were considered as cases, and 30 were age-matched controls depending on the selection criteria. 

Selection criteria

Inclusion criteria included all MBBS students who gave written informed consent and were not having any medical or psychiatric illness. Students who had undergone yogic training or heavy physical exercise for at least six months comprised the trained group and were considered as cases.

Exclusion criteria included subjects suffering from chronic debilitating diseases, such as cardiac arrhythmias, hypertension, diabetes, ischemic heart disease, retinopathy, nephropathy, respiratory diseases, and psychiatric illness; smokers, and those with a family history of metabolic diseases, including diabetes and hypertension; also, persons receiving any drug that may affect the autonomic reflexes and those who did not give consent and were not willing to participate in the study.

Data sources and measurement of variables

History was recorded, followed by an anthropometry and clinical examination of the subject. Body mass index (BMI) and body surface area (BSA) were calculated as per the standard formula. Resting pulse rate, blood pressure (BP), resting respiratory rate, physical ﬁtness index (PFI), breath holding time (BHT), and 40 mm Hg endurance test time (Flack's Air-Force Manometer Test) were measured.

The technique used for measuring breath holding time (BHT) [3] was explained and demonstrated to the subject first. They were asked to inhale maximally and then hold their breath till the breaking point was reached, i.e., the point when the subject could no longer hold their breath. The subject was motivated to maximize the breath-holding period. The time was noted in seconds by using a stopwatch.

For the 40 mmHg endurance test [4], the subject was asked to take a full breath and blow in the tube of the sphygmomanometer so that the mercury level rose up to 40 mm Hg. The subject was instructed to maintain the level as long as they could. The subject was continuously prompted to maintain the level and to prolong the holding period. The subject was also asked not to blow their cheeks while performing the test. For both the above tests, a minimum of three trials were given with a rest period of three minutes between the trials, and the highest of three similar best performances was taken for statistical analysis.

For measuring the maximal oxygen consumption (VO_2_ max) by the modified Harvard step test [4], the metronome was preset at a rate of 90/min. A wooden bench of 40 cm in height was used to first demonstrate the stepping cycle in rhythm with the step frequency to the subject. The duration of the test was set at five minutes. The subject was closely watched for any signs of discomfort or any indications to stop the test. Immediately after finishing the test, the subject's pulse rate was counted using the radial artery for a full minute, i.e., from 0-1 minute for VO_2_ max estimation by Astrand-Ryhming Nomogram. On the nomogram, the heart rate (0-1 minute) and weight in kilograms of the subject were accurately marked on their designated scales. A line was drawn between the two marks, and where this line intersects the VO_2_ max line in the middle, the reading was noted. After recording the pulse (0-1 minute) for VO_2_ max estimation, the radial pulse will again be counted at intervals of 1-1½, 2-2½, and 3-3½ minutes of completing the test to evaluate the physical fitness index (PFI). The PFI will be evaluated by using the following formula:

$$PFI=\frac{Duration\ of\ test\ in\ seconds}{2\times(Sum\ of\ pulse\ counts\ of\ 1-1\frac{1}{2},2-2\frac{1}{2},3-3\frac{1}{2}\ minutes)}\times 100$$

Statistical data analysis

The data was collected using the KoboToolbox application. Once the data was collected, it was tabulated, and statistical analysis was done using SPSS software version 16 (SPSS Inc., Chicago, US). The values of study parameters are presented as mean ± standard deviation (SD), and the means were compared using an unpaired Student's t-test. P-value <0.05 was considered significant.

## Results

Demographic data of study participants

The mean and standard deviation of demographic characteristics, including the age, height, weight, BMI (using standard formula), BSA in m^2^, pulse, respiratory rate, and blood pressure of all subjects (males and females), are presented in Tables 1 and 2. The difference and p-values between them were found. The mean age of control males and case males was 20.33 and 20.61 years, and the difference was not statistically significant (p=0.65). The difference in height between the two groups was also not statistically significant. The mean weight for the males in the case group was 59.80 kgs, and for the control group was 67.47 kgs, and the difference was statistically significant (p=0.022). Similarly, the mean weight for the females in the case group was 52.93 kgs, and for the control group was 62 kgs, and the difference was statistically significant (p=0.0378). Study participants who were engaged in regular physical activity had lower BSA values than the sedentary subjects. The mean pulse rates and resting blood pressure did not show any significant difference between the two study groups.

**Table 1:** Demographic characteristics of male subjects BSA - body surface area; RR - respiratory rate; BP - blood pressure

Parameters	Control males (n=16 )	Case males (n=20)	p-value
Age (years)	20.33±1.76	20.61±1.92	0.655
Height (cm)	169.13±7.48	172.2±6.34	0.191
Weight (kg)	67.47±11.33	59.80±10.43	0.023
BMI (kg/m^2^)	20.97±3.94	22.76±3.54	0.161
BSA (m^2^)	47.36±4.07	48.55±4.10	0.391
Pulse (beats/min)	82±7	80±13	0.584
RR (breaths /min)	18±4	20±5	0.202
Systolic BP (mm of Hg)	116±8	112±8	0.145
Diastolic BP (mm of Hg)	80±7	78±8	0.437

**Table 2:** Demographic characteristics of female subjects BSA - body surface area; RR - respiratory rate; BP - blood pressure

Parameters	Control females (n=14)	Case females (n=10)	p-value
Age (years)	20±0.78	19.56±1.09	0.260
Height (cm)	158.79±5.91	160.6±3.00	0.385
Weight (kg)	62±9.11	52.93±10.97	0.038
BMI (kg/m^2^)	21.00±4.14	24.06±5.07	0.118
BSA (m^2^)	44.97±4.71	43.82±3.63	0.525
Pulse (beats/min)	85±8	82±9	0.399
RR (breaths/min)	18±4	21±5	0.117
Systolic BP (mm of Hg)	113±8	110±6	0.328
Diastolic BP (mm of Hg)	76±10	73±8	0.441

Major outcomes and results

The values of cardiorespiratory efficiency parameters in males and females are depicted in Tables 3 and 4, respectively. The utmost precaution was taken to include those individuals who were within the same age range in both groups for both sexes. This was done to exclude any age-related confounding factors which could have had an effect on cardiorespiratory efficiency parameters.

**Table 3:** Values (mean ± SD) of cardiorespiratory efficiency parameters in males PFI - physical ﬁtness index; BHT - breath holding time; VO_2_ max - maximal oxygen consumption

Parameters	Control males (n=16)	Case males (n= 20)	p-value
PFI (%)	88.82±5.56	96.05±7.44	0.002
40 mmHg ET (sec)	36.47±8.45	48.88±8.64	0.0001
BHT (sec)	44.80±14.18	58.91±12.35	0.0031
VO_2_ max (liters/min)	2.41±0.58	3.6±0.90	0.0001

**Table 4:** Values (mean ± SD) of cardiorespiratory efficiency parameters in females PFI - physical ﬁtness index; BHT - breath holding time; VO_2 _max - maximal oxygen consumption

Parameters	Control females (n=14)	Case females (n=10)	p-value
PFI (%)	82.06±8.95	96.55±6.47	0.0003
40 mmHg ET (sec)	27.79±10.30	38.4±10.69	0.0227
BHT (sec)	42.29±15.37	54.60±13.36	0.0536
VO_2_ max (Litres/min)	2.14±0.49	3.76±0.69	0.0001

**Figure 1:**
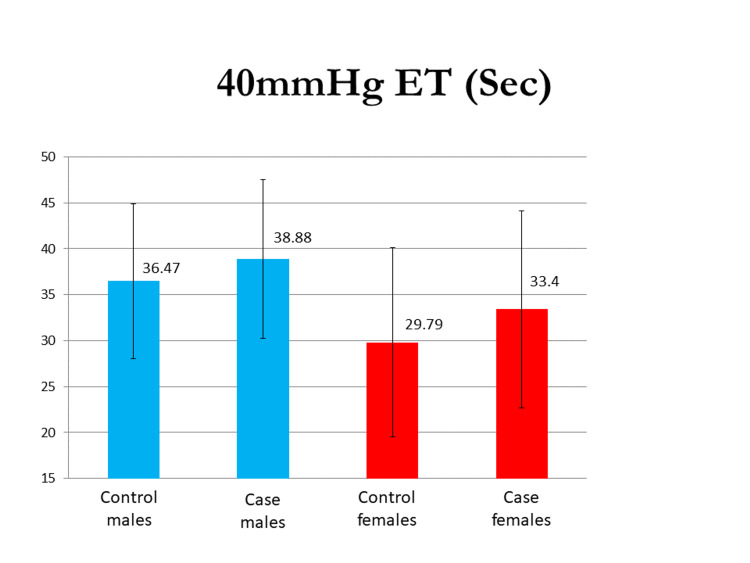
A graphical representation of the results of the 40 mm Hg endurance test in both males and females

**Figure 2:**
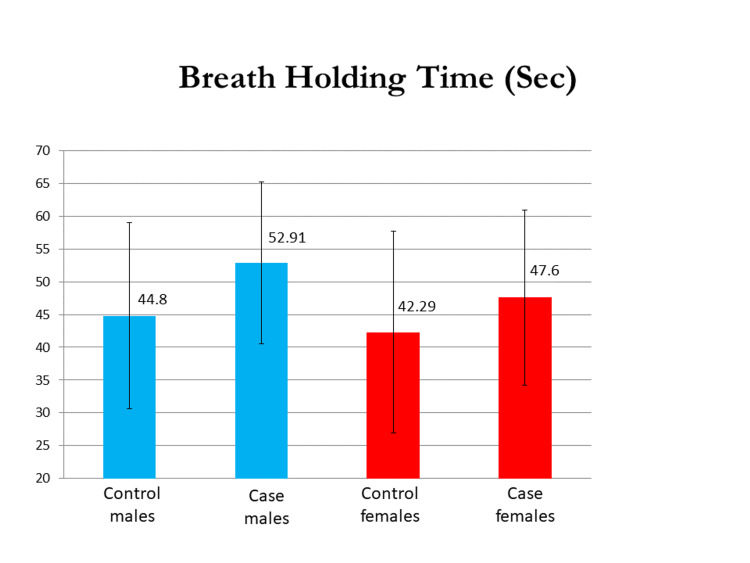
A graphical representation of the results of the breadth holding time in both males and females

**Figure 3:**
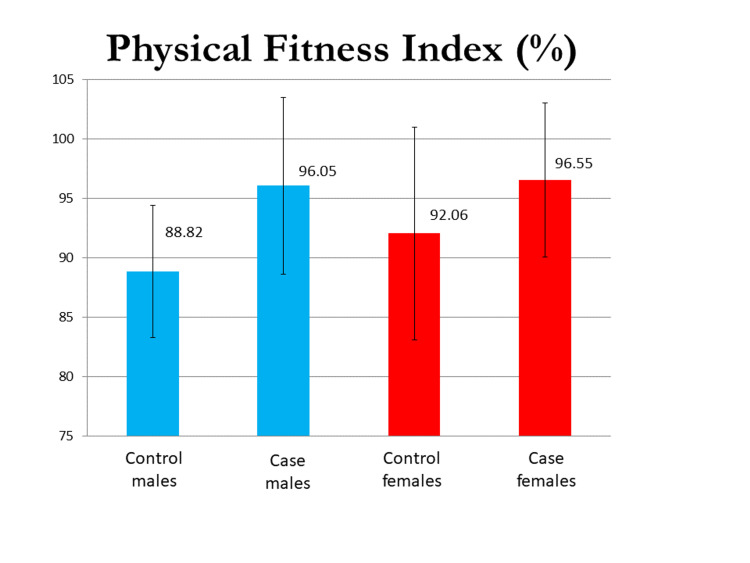
A graphical representation of the results of the physical fitness index in both males and females

**Figure 4:**
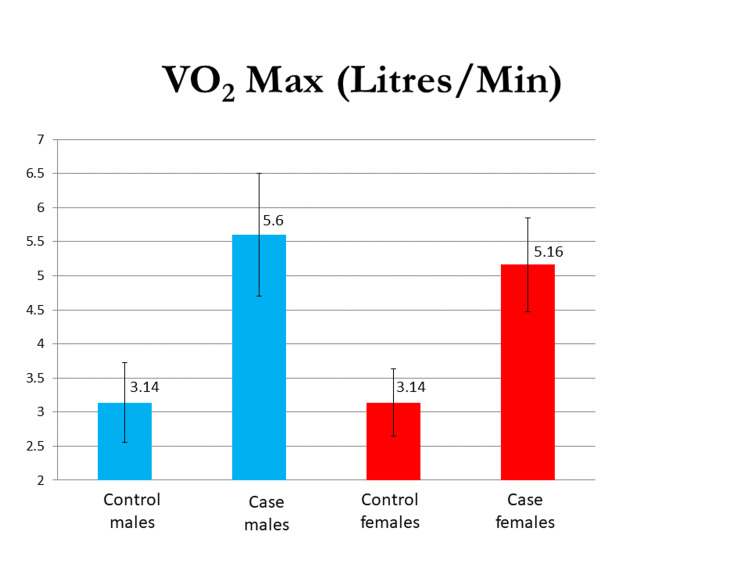
A graphical representation of the results of the VO2 max in both males and females VO_2_ max - maximal oxygen consumption

The mean 40 mm Hg endurance test (in seconds) in the male group was 36.47±8.45 and 48.88±8.64, and in the female group, it was 29.79±10.30 and 38.4±10.69 in the control and case groups, respectively. The VO_2 _max evaluated by the modified Harvard step test was 2.41±0.58 L/min in the control males group and 3.6±0.90 L/min in the case group of males, and 2.14±0.49 L/min in the control group of females and 3.7 ±0.69 L/min in case group of females. The difference in their means was statistically significant (p<0.0001). The mean PFI (%) in males was 88.82±5.56 and 96.05±7.44 and that in females was 82.06±8.95 and 96.55±6.47 in the control and test groups, respectively, and the difference was significant in both sexes. Mean BHT (in seconds) in males was 44.80±14.18 and 58.91±12.35 and that in females was 42.29±15.37 and 54.60±13.36 in control and case groups, respectively, with a p-value of <0.05 in both sexes. Graphical representations of the results of the 40 mm Hg endurance test, breadth holding time, physical fitness index, and VO_2_ max in both males and females are seen in Figures 1-4.

## Discussion

The purpose of this study was to determine the effect of yogic practice on the cardiovascular response to exercise endurance and physical fitness. Physical fitness depends mainly on the cardiorespiratory endurance of an individual. Yoga practice has proved to be beneficial in maintaining a physiological milieu pertaining to cardiovascular parameters [5]. The results of the evaluation of VO_2_ max indicate that all the physically untrained subjects had a VO_2_ max value of less than 2.41 l/kg/min, while all the subjects practicing yoga had a VO_2_ max of more than 3.76 l/kg/min. The VO_2_ max was found to be significantly higher in the case group in the present study. The findings in our study are in accordance with a previous study by Gupta et al. [6], who have also assessed BMI, BSA, resting respiratory rate, pulse rate, BP, VO_2_ max, and PFI. They reported that regular exercise training increases VO_2_ max and PFI and decreases resting pulse rate and blood pressure. They also showed a good negative correlation between BMI and VO_2_ max. An earlier study by Banerjee et al. [7] on 70 normal healthy Indian Air Force Personnel reported their mean absolute VO_2_ max values around 2.5 l/min, which corroborates with our values of the control group.

A study by Lim and Lee [8] was conducted in the Singapore armed forces to study the effects of a 20-week basic military training program on VO_2 _max and the aerobic fitness of obese recruits. The results indicate that the VO_2_ max of the subjects significantly increased after a 20-week training program. It could be attributed to the fact that rigorous training in the army improves VO_2_ max significantly. In the present study, the results of the PFI indicate that most subjects in the control group fell in the category of 70 to 100%, and no subject had an index of more than 100%, while one individual from the case group even had a PFI of more than 100%. PFI of >96% is rated as excellent, 83-96% as good, 68-82% as average, 54-67% as below average, and <54% is considered poor [9].

The positive effects of yoga may be mediated by increased vagal activity and decreased cortisol. Vagal activity has been found to increase significantly after practicing yoga. This likely happens via stimulation of dermal and/or subdermal pressure receptors that are innervated by vagal afferent fibers, which ultimately project to the limbic system, including hypothalamic structures involved in cortisol secretion. These pathways are supported by anatomical studies indicating that baroreceptors and mechanoreceptors within the dermis (i.e., Pacinian corpuscles) are innervated by vagal afferent fibers. Second, functional studies have indicated that electrical vagal stimulation results in reduced cortisol [10]. Yoga has also been noted to lead to decreased cortisol [11]. A study by Darr et al. [12] indicated that trained subjects demonstrated a significantly faster heart rate recovery as compared to untrained subjects. This could be a reason for higher PFI as well. 

In the present study, the results of the breath-holding test also indicate that people from the case group had more BHT compared to the control group. The values for BHT were 52.40 after training and 45.75 before training, according to Joshi and Joshi [13]. On the other hand, Madanmohan et al. [14] recorded BHT of 89.07 after yoga training and 63.89 before yoga training. Findings from the present study are in line with this study.

Our results with parameters like BHT and 40 mmHg endurance test are also similar to the observations of O'Sullivan and Bell [15], who have reported that physical training blunts the pressor, tachycardiac and vasodilator responses, and they attributed this to blunting of sympathetic vasodilator activation.

Bera and Rajapurkar [16] have reported that yoga training results in significant improvement in cardiovascular endurance. This is consistent with the fact that yoga training improves physical efficiency as measured by the Harvard step test. Yoga training increases muscular endurance, delays the onset of fatigue, and enables one to perform work at lesser VO_2_ max [17]. However, in contradiction to this, when Beutler et al. [18] tested whether yogic subjects show an increased endurance capacity compared to matched non-yogic individuals with similar physical activity levels, their results proved that yoga practice was not associated with improved exercise capacity nor with significant changes in exercise ventilation despite a significantly different respiratory regulation.

Accelerated cardiovascular response to exercise is known to be a risk factor for cardiovascular disease, whereas reduced reactivity is an indicator of fitness [19]. An increase in PFI (in males) and VO_2_ max in both sexes was observed in this study which is suggestive of a reduction in metabolic demands on the cardiac muscle, sparing the heart from undue exertion and helping increase the cardiac output when required (as during yoga exercises). So, yoga is very effective in improving health, especially cardiorespiratory fitness [20]. These positive physiological outcomes of yoga have physiological significance as well as clinical applications and important implications in the prevention of cardiovascular disease.

In line with any research endeavor, this study also had a couple of limitations. It was a pilot study, and due to time constraints, the study duration was only six months. Moreover, an intervention wasn't employed as an interventional study was to be planned later as per the outcomes of this pilot project.

## Conclusions

By studying the dynamics of the various cardiorespiratory responses, we have determined the values of fitness parameters in subjects involved in yogic practices. Cardiorespiratory efficiency parameters were found to be evidently better in youths who have undergone a specific duration of yogic exercises. It was found that the yoga group had statistically significantly higher VO_2_ max (p<0.001) and better PFI, BHT, and 40mm Hg endurance test values than the control group. Thus, the results of the present study indicated that yogic practice caused substantial conditioning of cardiorespiratory parameters and can improve the aerobic capacity of the individual.
